# Pharmacokinetics and pharmacodynamics of esomeprazole and ranitidine during continuous venovenous hemodiafiltration

**DOI:** 10.1186/cc12373

**Published:** 2013-03-19

**Authors:** V Fuhrmann, P Schenk, W Jaeger, M Miksits, R Kitzberger, U Holzinger, C Madl

**Affiliations:** 1 Medical University Vienna, Austria; 2 Landesklinikum Thermenregion Hochegg, Grimmenstein, Austria; 3 University Vienna, Austria; 4 Kaiser Franz Josef Krankenhaus, Vienna, Austria; 5 Krankenanstalt Rudolfstiftung Wien, Vienna, Austria

## Introduction

Esomeprazole, a proton pump inhibitor, and ranitidine, a histamine-2 receptor antagonist are frequently used in intensive care patients requiring stress ulcer prophylaxis. Continuous venovenous hemodiafiltration (CVVHDF) is an important extracorporeal renal replacement therapy in critically ill patients suffering from multiple organ failure. This study investigates the pharmacokinetics and pharmacodynamics of esomeprazole and ranitidine in anuric critically ill patients undergoing CVVHDF.

## Methods

Esomeprazole 40 mg was administered intravenously once daily in nine intensive care patients and ranitidine 50 mg was administered intravenously three times daily in nine intensive care patients with acute renal failure undergoing CVVHDF who required stress ulcer prophylaxis. The concentration of esomeprazole and ranitidine in serum and ultradiafiltrate was determined by high-performance liquid chromatography. The intragastric pH was obtained via intragastric pH-metry.

## Results

The mean peak prefilter concentration, AUC_0-24_, volume of distribution, half-life, total clearance and hemodiafiltration clearance of esomeprazole were 6.9 μmol/l, 30.6 μmol.hour/l, 53.3 l, 2.0 hours, 1.4 l/hour and 0.2 l/hour, respectively. In the ranitidine group the mean peak prefilter concentration, AUC_0-8_, volume of distribution, half-life, total clearance and hemodiafiltration clearance were 5.5 μmol/l, 12.0 μmol. hour/l, 91.4 l, 14.4 hours, 5.8 l and 1.3 l, respectively. Median time of intragastric pH >5 and median intragastric pH decreased significantly in the ranitidine group over time (*P *0.05) but not in the esomeprazole group.

## Conclusion

Although pharmacokinetics of esomeprazole and ranitidine are only little influenced by CVVHDF, we found a significant decrease of intragastric pH in patients treated with ranitidine. Esomeprazole may be superior for long-term stress ulcer prophylaxis in critically ill patients undergoing CVVHDF.

